# Impact of Intercalated Degree on Post-graduate Career Progression and Academic Development in the UK: A Rapid Review of the Literature

**DOI:** 10.7759/cureus.24569

**Published:** 2022-04-28

**Authors:** Mohammed El-hassan

**Affiliations:** 1 Exeter Medical School, University of Exeter, Exeter, GBR

**Keywords:** medical students, academic achievements, intercalated degree, career progression, post-graduate

## Abstract

An intercalated degree offers medical students an avenue to explore their interests and become competent in scientific literacy. Although intercalating can pose a financial burden and time commitment, they do provide competitive academic and speciality training applications. The aim of this review was to explore academic development and subsequent outcomes of career progression. Medline and EMBASE were systematically searched using keywords. After the removal of duplicates, the studies were screened against the inclusion criteria. For the five studies included in this review, a narrative synthesis was performed. The two main themes were academic development and career progression. All studies showed a plethora of academic achievements during and after intercalation. Two studies showed that students are more likely to enter a career in academic medicine. A further two studies have shown that the transferable skills of academia have allowed alumni to make more competitive applications for foundation year and speciality training. The results have shown a correlation between academic achievements and an increase in competitiveness in foundation programmes and speciality applications. There are clear discrepancies in the success of academic careers depending on the institution and type of intercalated degree. Current literature suggests a master’s degree results in more academic success compared to a bachelor’s degree. Due to the sheer diversity of intercalated degrees offered to medical students in the UK, there is limited literature on post-graduate career progression. More research should be undertaken to look at the implications of intercalation on post-graduate career progression.

## Introduction and background

The General Medical Council aims to graduate well-rounded clinicians, but with a tightly bound undergraduate medical curriculum, it is difficult to acquire the skills of scientific literacy, self-directed work and exploring areas of interest [1,2]. However, an additional degree has the potential to meet many of these objectives [3].

Intercalated degrees are offered in a variety of subjects at both undergraduate (BSc, BA or a BMSc) and postgraduate (MSc, MRes) levels, to be conducted during medical school. Intercalated degrees are incentivised for competitive academic and speciality training applications, as well as better performance in medical school subsequently [3-5]. However, since the rise in tuition fees in 2012, students have considered an extra degree to be a financial burden [6]. Therefore, to widen participation, doctors graduating as of 2023 can no longer gain additional points on the UK Foundation Programme (UKFP) application [7]. This rapid review aims to update the literature from the previous systematic review conducted by Jones et al. in 2013, to explore academic development and subsequent outcomes of career progression.

## Review

Methods

Search Strategy

The framework of The Preferred Reporting Items for Systematic Reviews and Meta-Analyses (PRISMA) guidelines [8] was used to ensure methodological rigour. Medline and EMBASE were used to search for relevant literature, on the OvidSp platform on October 13, 2021. The search terms of the Medline and EMBASE search are outlined in Table 1. Forward and backward citation chasing of identified articles were found using WebOfScience (n=1). Duplicates were then removed.

**Table 1:** Search terms used

	Terms searched
1	'medic* student*'.ti,ab.
2	'student doctor*'.ti,ab.
3	intercalat*.ti,ab.
4	msc.ti,ab.
5	bsc.ti,ab.
6	progress.ti,ab.
7	career.ti,ab.
8	training.ti,ab.
9	research.ti,ab.
10	outcome.ti,ab.
11	postgraduat*.ti,ab.
12	1 or 2
13	3 or 4 or 5
14	6 or 7 or 8 or 9 or 10 or 11
15	12 and 13 and 14

Inclusion Criteria

After the duplicates were removed, the following inclusion criteria were set to screen the titles and abstracts of the remaining articles (n=90):

· Studies published in the English Language

· Studies published in the last 10 years

· Studies conducted in UK medical schools

**Figure 1:**
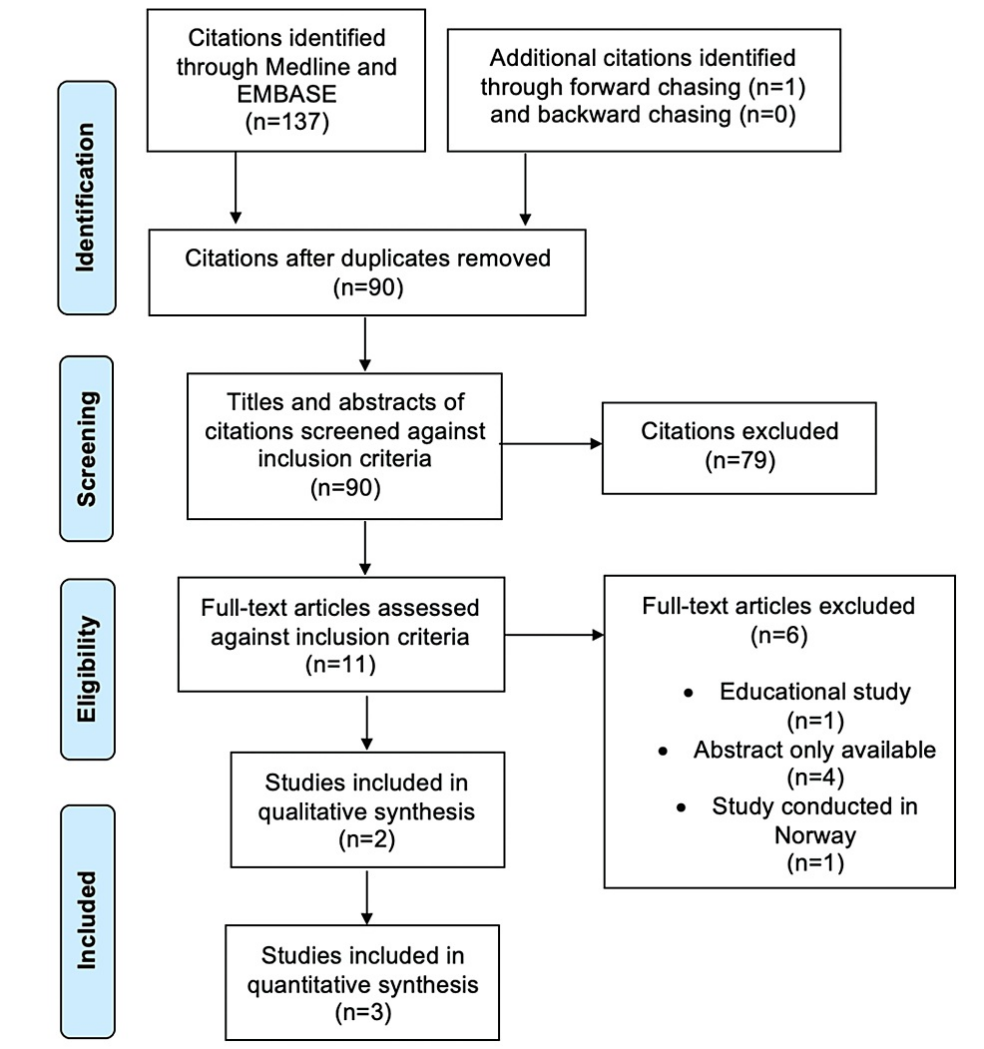
PRISMA flowchart

Figure 1 illustrates the PRISMA flowchart.

Data Extraction

A data extraction form was created, to ensure relevant information was obtained from the five studies. Table 2 illustrates the full data extraction form.

**Table 2:** Data extraction form

	Content extracted from studies
1	Title
2	Author(s) & year published
3	Journal
4	Aim of study
5	Study design
6	Country study carried out in
7	Type of participants (i.e. role)
8	Number of participants
9	Response rate
10	Number of years since intercalation (*alumni only)
12	Brief description of participant demographics
12	Criteria being assessed/reviewed
13	Results
14	Conclusions
15	Related references that could be useful
16	Other comments
17	MERSQI/CASP (quality appraisal)

Synthesis and Quality Assessment

A narrative synthesis was performed. The methodological quality of the quantitative studies was evaluated using the Medical Education Research Study Quality Instrument (MERSQI) [9]. In parallel, the methodological quality of the qualitative studies was evaluated using The Critical Appraisal Study Programme (CASP) [10]. MERSQI score was modified as various items were not applicable. Using the same calculation as several studies have done, the score was converted back to out of 18for standardisation [11,12]. There is no formal cut-off for a good score. However, papers in the past have used 14 as a good cut-off score [13,14]. Based on the criterion from previous literature, the studies are not classified as high quality. The CASP tool used 10 questions to screen for the robustness of the qualitative studies used.

Results

Table 3 provides an overview of the five studies used in this review.

**Table 3:** Overview of the studies used BMSc - Bachelor of Medical Sciences, BSc - Bachelor of Sciences, MRes - Master of Research, CASP - Critical Appraisal Skills Programme, MERSQI - Medical Education Research Study Quality Instrument

Author(s) and year published	Number of participants	Type of intercalating degree and medical school	Response rate	Career status A = Intercalating B = Intercalated (but still in medical school) C = Alumni	Study Design A - single group cross-sectional B - single group post-test only C - exploratory case study	Outcomes measured A - academic development B - career progression	Key findings	Quality assessment
Graham et al., 2019 [15]	37	BSc in Urgent and Emergency Care - Plymouth	80%	B + C	A	A + B	Students involved in research had high publication rates. Academic, clinical, and inter-professional skills developed	MERSQI (9/18)
Sorial et al., 2021 [4]	52	Master of Research (MRes) - Manchester	68%	C	B	A + B	73% of alumni career progression had taken minimum number of years Majority completed academic posts	MERSQI (12/18)
Stubbs et al., 2012 [6]	166 - Bristol 94 - Sheffield	Not specified - Bristol and Sheffield	52.5% - Bristol 58.7% -Sheffield	B	A	A	Sheffield intercalators had more successful academic outcomes. Intercalation was perceived to increase chances of an academic job.	MERSQI (12/18)
Muir et al., 2020 [16]	10	BMSc Medical Education - Dundee	23.8%	B + C	C	A + B	Significant improvement in critical appraisal, reflection, and independent organisation.	CASP 1-9: YES 10: Moderate
Muir et al., 2014 [5]	13	BMSc Medical Education - Dundee	100%	B	C	A	Personal development. Students valued the application that intercalating had on their future practice and career development.	CASP 1-3, 5, 9: YES 4,8: NO 10: Moderate

Following the analysis of the literature, two main themes were found: academic development and career progression.

Academic Development

Academic achievements during and following the intercalated degree include publications, presentations at conferences, research grants and further post-graduate qualifications [4,6,15]. Several studies from clinically orientated and academic intercalated degrees found students who conducted research had high publication rates [4,15]. Moreover, perceived academic outcomes have included improved critical appraisal and research skills [5,16].

Career Progression

Career outcomes were divided into two categories: perceived career outcomes while undertaking the intercalated degree and career outcomes as alumni post-graduation. Stubbs et al. found that students from numerous institutions undertake an intercalated degree with the perception that it will increase their chances of securing an academic job in the future [6]. Studies show that students who pursue a research or clinically orientated intercalated degree are more likely to enter a career in academic medicine [4,15]. Sorial et al., in 2021, found that career progression for alumni took the minimum number of years, using the NHS career algorithm [4]. 63% of participants on this programme had completed an academic component of their post-graduate training such as Academic Foundation Programme (AFP) and Academic Clinical Fellowship (ACF) [4]. However, in other studies, some students used the degree as an opportunity to gain transferable skills for their future career path [5,16]. The transferable skills of academia have allowed alumni to have more competitive applications for foundation year and specialty training [4,16].

Study Quality

All studies were single-centred, except for one multi-centred study, which compared two institutions [6]. The mean response rate was 63.8% (range=23.8%-100%). The mean MERSQI score was 11 (range=9-12). For the qualitative papers, using the CASP checklist, all studies have robust methodology, although small sample sizes were recorded in both studies.

Discussion

The results have shown a correlation between academic achievements and increase competitiveness to foundation programme and speciality applications. Although UKFP is no longer accepting additional points from publications and intercalated degrees, there is increased competitiveness for speciality training [1,4].

The ability to reflect and improve in self-directed learning are invaluable attributes that are encouraged for clinicians [17], many of which students in Muir and Law study in Dundee have developed [5]. Students perceived career outcomes included being 'more likely to go into academic medicine', although only two of the 13 students within the cohort were interviewed for AFP. However, the small sample size is arguably not indicative of the overall number of students that had studied Medical Education.

Studies have found that intercalation greatly increases the chances of pursuing a related career [4,15]. Stubbs et al. compared institutions academic achievements during intercalation [6]. Sheffield was found to produce significantly more posters and publications compared to Bristol, although most students in Sheffield completed a BMSc whereas in Bristol a BSc. With perceived career outcomes, 29.1% of students in Sheffield and 46.3% of students in Bristol undertook an intercalated degree with the ambition of improving their chances of securing an academic job. Data were comparable as students from both institutions had similar demographics. However, as this was a retrospective study, it is subject to recall bias. Thus, a follow-up would be useful to evaluate the number of students who secured an academic post following graduation.

Sorial et al. had looked at this approach with alumni from the MRes at Manchester University [4]. Alumni were assessed three to four years post-graduation. A plethora of competitive academic posts were secured by the alumni in their early career stages. To assess the career outcomes effectively, there must be a sufficient follow up time given post-graduation. Three to four years may be a sufficient time to assess early career progression, though longevity is an area that should be explored.

There are clear discrepancies in success of academic careers depending on the institution and type of intercalated degree [1,6]. Although you cannot generalise the outcomes of all intercalated degrees from these studies, current literature suggests a master’s degree result in more academic success compared to a bachelor’s degree [1]. As academic success is correlated with increased competitiveness for speciality training [18], a master's is arguably more beneficial.

Although students show a career interest in their chosen intercalated degree [4-6,15,16], students undertaking the degree naturally bring pre-existing interest in the area. Moreover, students who intercalate in general, particularly with a master’s degree, tend to be more motivated academically [1]. Therefore, with or without the intercalated degree, they may have similar outcomes post-graduation. These confounding variables make it difficult to attribute career outcomes solely to the intercalated degree [15].

Lastly, four out of the five studies were single centred and included only one intercalated degree [4,5,15,16]. Therefore, although there is a plethora of intercalated degrees offered by forty medical schools in the UK [19], current data is not representative, hence there is limited evidence on the career progression of alumni following graduation.

Future study recommendations

Multi-centred, or single-centred studies with numerous intercalated degrees, would need to be undertaken. A comparative study from a group of intercalators and non-intercalators from the same institution would be a useful comparative analysis of post-graduate progression. These proposed studies will allow students to make more informed decisions.

Strengths and limitations

This review pertains to an extensive search strategy. The use of the CASP and MERSQI criteria has allowed the standardisation of quality appraisal of each article included in the results. Although limited literature were identified in career progression of alumni [4,16], they did align with a bigger body of evidence from the systematic review conducted in 2013 by Jones et al. [3].

## Conclusions

Intercalated degrees aid medical students in diversifying their academic portfolios both during and after medical school. The progression in academia is linked with an increase in success with competitive applications for speciality training post-graduation. Moreover, students are more confident with their transition to being junior doctors due to the transferable skills that they had gained during intercalation. Due to the sheer diversity of intercalated degrees offered in the UK, more research should be undertaken to look at the implications for post-graduate career progression.
